# Effectiveness of an artificial intelligence-based system to curtail wind turbines to reduce eagle collisions

**DOI:** 10.1371/journal.pone.0278754

**Published:** 2023-01-26

**Authors:** Adam E. Duerr, Amy E. Parsons, Laura R. Nagy, Michael J. Kuehn, Peter H. Bloom

**Affiliations:** 1 Bloom Biological, Inc., Santa Ana, California, United States of America; 2 Avangrid Renewables, LLC, Portland, Oregon, United States of America; 3 Bloom Research, Inc., Santa Ana, California, United States of America; San Diego Zoo Institute for Conservation Research, UNITED STATES

## Abstract

Operating wind-power projects often includes protecting volant wildlife. One method for doing this uses an automated system to detect, identify (through use of artificial intelligence; AI), track animals (targets) and curtail turbines when risk of a collision is high. However, assessments of the effectiveness, in terms of identification accuracy and subsequent turbine curtailment of such systems are lacking. Over 1 year, we assessed such an automated system installed at a wind project in California, USA to determine its identification accuracy and rates at which "virtual” curtailments were ordered (without slowing turbines), for eagles (intended targets) and non-eagle targets. The system correctly identified 77% of eagles and 85% of non-eagles. Curtailment orders occurred 6 times more frequently for non-eagle targets (5,439) than for eagle targets (850). Greater abundance of common ravens that were misidentified as eagles influenced the effectiveness of the system by greatly increasing unintended curtailment orders. The balance between costs (price of the IdentiFlight system, reduced energy generation, turbine wear and maintenance) and benefits (reduced collisions between intended target species and turbines) may depend upon the biological setting, speed at which operators can curtail turbines, and the objectives of the operator when considering the IdentiFlight system.

## Introduction

In many parts of the world, operating a wind-power project includes minimizing risk of protected volant wildlife from colliding with wind turbine blades [1–5]. One method of minimizing such risk is to install systems that detect wildlife, use artificial intelligence (AI) to identify protected species, and initiate some action intended to minimize collision risks (e.g., provide visual or auditory signal, curtail turbines). While there have been limited assessments of such systems [6–10], there is a general lack of information that can be used by wind-power operators to assess how curtailments could potentially influence costs as they relate to identification accuracy and subsequent turbine curtailment from use of AI-based systems (but see [3]).

The decision to implement a technological solution to minimize risk to wildlife is dependent on the effectiveness of the technology and the associated costs of implementation and operation of that technology. If the technology is effective, it can provide the benefit of reducing the financial liability associated with operational-related fatalities of protected species [11]; however, there are legal and cost implications when a technology system fails to curtail for a protected species (intended target) that was misidentified. Thus, in these technology systems, effectiveness and costs are often interrelated because every turbine curtailment incurs costs due to reduced power production and increased wear and maintenance of individual turbines. These costs occur both for curtailments implemented to protect designated species (intended targets) and implemented for misidentified species (unintended targets). Specifically, the use of AI-based systems to initiate curtailments has costs that come in the form of costs of both curtailing (i.e., effective with associated costs) and failing to curtail turbines (i.e., ineffective with associated costs). The first steps in assessing these costs are to identify rates at which protected species are accurately identified and misidentified by the AI software and, in turn, the rates that curtailments are issued for intended and unintended targets.

**Fig 1:**
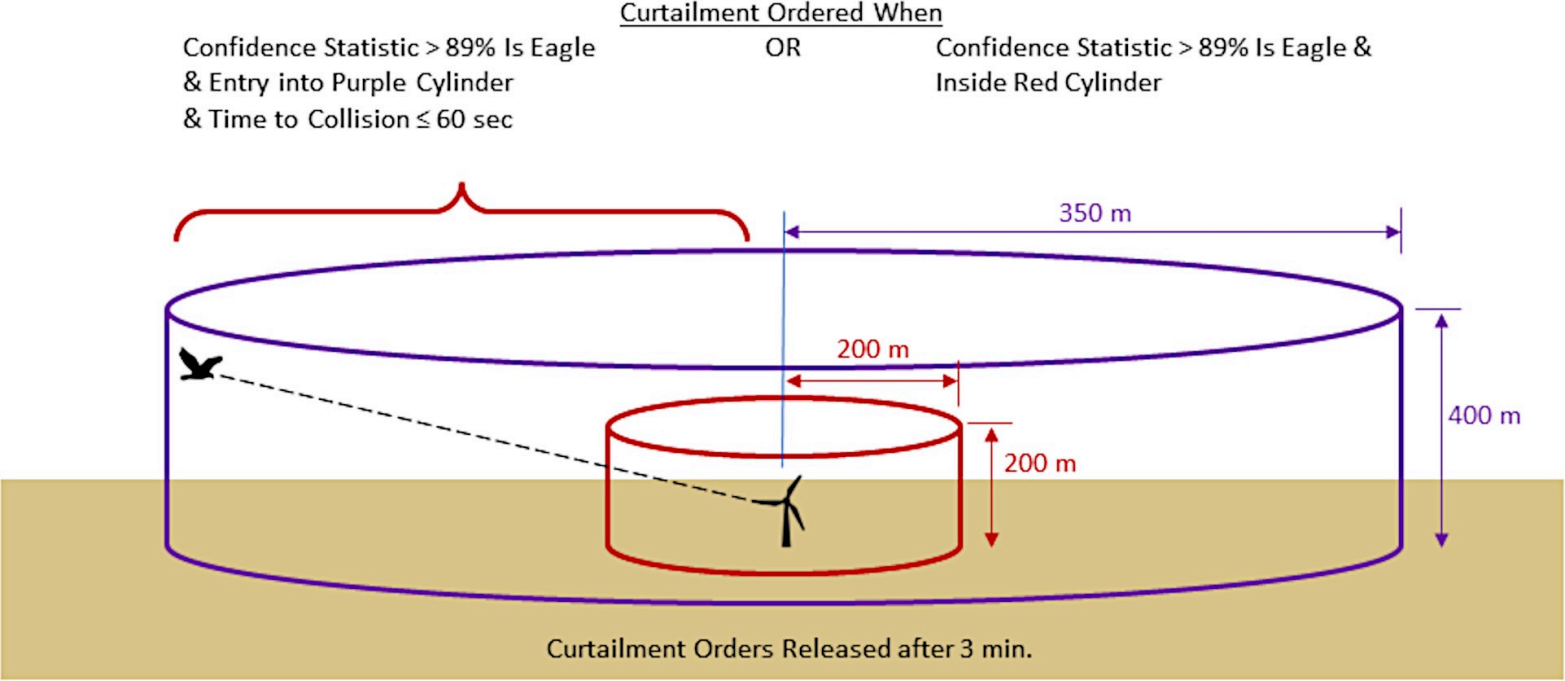
Illustration of the curtailment prescription for the IdentiFlight system installed at Manzana Wind Power Project, California, USA. Curtailments were ordered when the confidence statistic that a target was an eagle > 89% and either the target was within the purple cylinder and heading toward rotor-swept zone (RSZ) such that time to collision ≤ 60 s or the target was within the red cylinder. Curtailments were released 3 min after conditions were no longer met.

One AI-based system, IdentiFlight, has been installed at locations in North America, Europe, and Australia and has been associated with reduced mortalities of intended targets (eagles) in one location where it was assessed [7, 8]. This system incorporates a network of tower-mounted cameras and computers to detect, identify, and track avian species up to 1000 m from the cameras (see S1 File). Once a target is detected, the IdentiFlight system uses two AI processes to identify that target. The first uses feature-based attributes (e.g., estimated sizes, colors, orientation) to identify targets by comparing feature sizes (e.g., body length, wingspan) to sets within a classification table to determine the target identification. The second process uses a convolutional neural network to identify targets based on images of known species. Both AI processes assign confidence probabilities to each identification. This confidence probability, along with the location and trajectory of a target relative to a wind turbine, is used to determine when the system issues a curtailment of a wind turbine based on a curtailment prescription (Fig 1).

We evaluated the effectiveness of the IdentiFlight system to curtail wind turbines to reduce the risk of golden eagles colliding with them. For this evaluation, we 1) determined the rates at which the system correctly identified eagles, misidentified eagles as non-eagles (false-negative error), and misidentified non-eagles as eagles (false-positive error). We then used these to 2) determine the rates at which curtailment orders were effective or ineffective. Effective curtailments are those in which a curtailment is issued for an eagle at risk of colliding with a turbine (come near or enter rotor-swept-zones, RSZ, of turbines) whereas ineffective curtailments include both issuing a curtailment for eagles not at risk and for issuing a curtailment for non-eagles.

## Methods

### Study area

**Fig 2:**
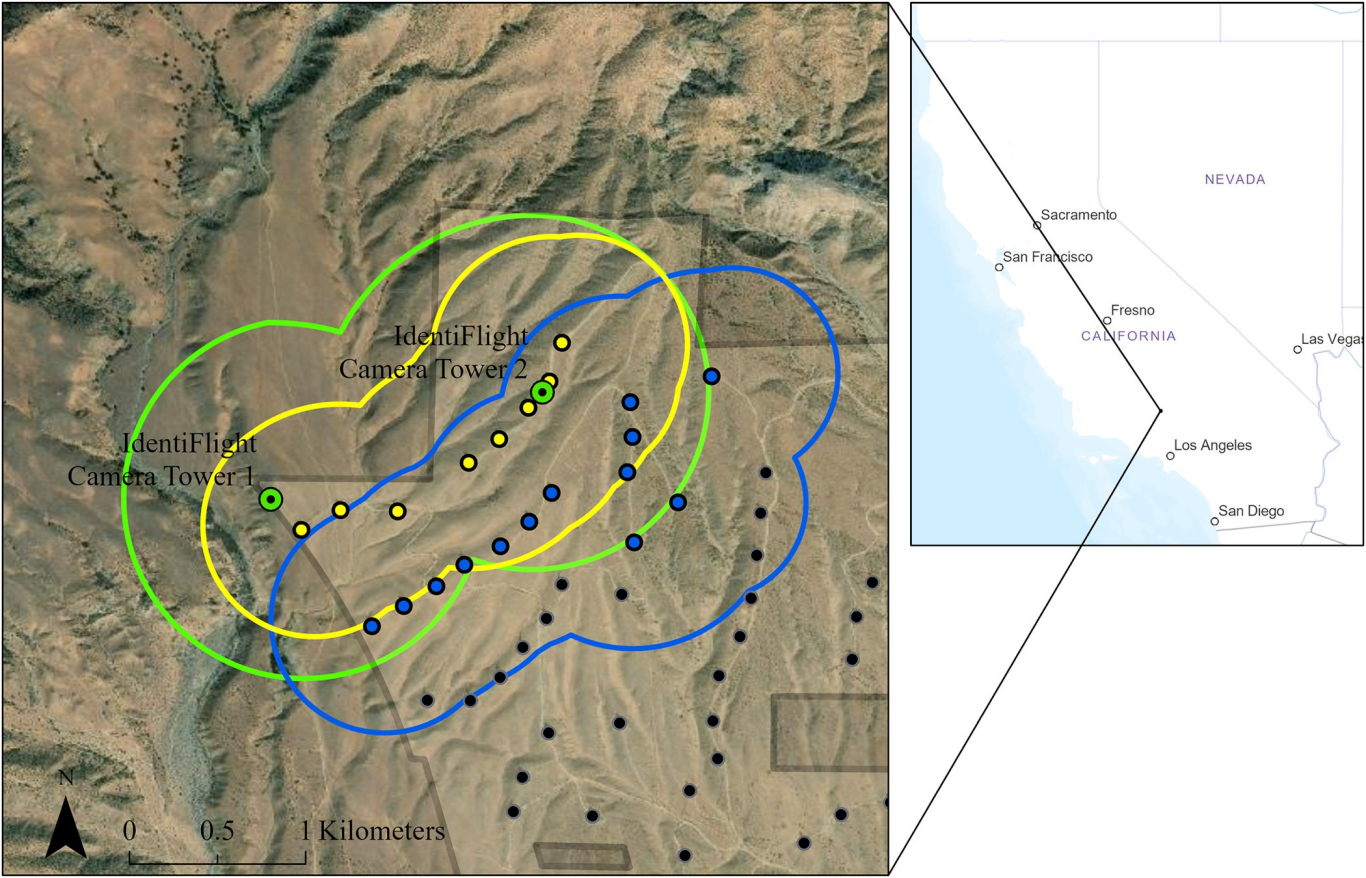
IdentiFlight system cameras (green dots) and turbine locations including those fully covered (yellow dots) and partially covered (blue dots) by the IdentiFlight system along the northern portion of the Manzana Wind Power Project, California, USA. Turbines were fully covered when 600 m around them (yellow line for fully covered turbines, blue line for partially covered turbines) was within 1000 m (green line) of an IdentiFlight camera.

We evaluated an IdentiFlight system from 21 June 2018 to 20 June 2019 installed at the Manzana Wind Power Project, in Kern County, California, USA. The project is a 189 MW wind energy facility consisting of 126 General Electric SLE 1.5 MW turbines, each with a hub height of 65 m, rotor diameter of 77 m, and maximum blade-tip height of 103.5 m. Turbines are located at elevations ranging from approximately 977 to 1,320 m above mean sea level and arranged in multiple strings oriented roughly southwest to northeast. The facility is adjacent to the southern foothills of the Tehachapi Mountains in the western portion of the Mojave Desert. The IdentiFlight system includes two camera units installed on 8 and 9 m towers, which were located at the highest elevation of the project where golden eagle activity was highest (personal observations, PHB) (Fig 2). The specific locations of the towers were chosen to maximize coverage of wind turbines by the two-tower system. Access to the study area was provided by Avangrid Renewables, LLC. Permits for animal research were not required because the methods described below were observational and did not require capture or handling of animals.

### Identification accuracy

The IdentiFlight system archives data on identification of targets, confidence of identifications, three-dimensional location of targets, and distance from targets to individual turbines and camera towers (see S1 File). These archives incorporate records collected about once per second, which usually include an image of the target, and are organized into tracks to identify repeated measures of the same target. The IdentiFlight base station then processes these data and can compare conditions to a curtailment prescription that is defined by a project operator’s goals and constraints to determine if curtailment of a turbine should occur (Fig 1). Curtailment decisions can then be passed from the base station to the Supervisory Control and Data Acquisition (SCADA) system at the wind-power project to control specific turbines. For this project, “virtual” curtailments were archived; however, wind turbines were not slowed or stopped by the IdentiFlight system (virtual curtailments and curtailment orders are synonymous).

To determine the accuracy of the IdentiFlight system to identify eagles, we compared the identification made by the system to an independent (blind) determination made by raptor biologists for each track. The raptor biologists used two designations, eagle and non-eagle. The non-eagle designation included all birds that were not an eagle species, inanimate objects (e.g., turbine blade, plane, balloon), and any target that could not be classified definitively as an eagle based on images recorded by the IdentiFlight system. All images within each track were reviewed separately by two raptor biologists to determine the target identity for each track. Targets that were designated as an eagle by both initial reviewers were recorded as such. For targets that were designated as an eagle by one raptor biologist but as a non-eagle by the other, a third raptor biologist reviewed the images to make a final determination. We then compared the identification made by the IdentiFlight system to the determination made by raptor biologists and classified each IdentiFlight record as correct, as a false-negative error (IdentiFlight misidentified an eagle as a non-eagle), or as a false-positive error (IdentiFlight misidentified a non-eagle as an eagle).

There were several situations in which we did not include some records from the IdentiFlight system in our analysis. We removed records from tracks when more than one unique target was captured by system images contained within a track. This situation arose when a single record (image) contained more than one bird. Only data from the first target within a track was retained for further consideration. We also removed tracks in which the IdentiFlight system identified a target but did not record an image of that target.

We calculated accuracy and error rates made by the IdentiFlight system separately for each of five time periods among which the IdentiFlight system changed throughout the study (Table 1, S2 File). Major changes to the system included improvements to identification algorithms and corrections for equipment failures that could have affected target identification. We analyzed data from each IdentiFlight tower separately because each tower identified targets independently (i.e., both towers could have detected the same target and identified it differently, although we did not quantify this) and operated in different terrain settings (Fig 2). We calculated accuracy rates as the number of correct identifications by the IdentiFlight system divided by the total number of records analyzed. The false-negative rate is the number of false-negative errors divided by the number of records in which the target was determined to be an eagle. The false-positive rate is the number of false-positive errors divided by the number of records in which the target was determined to be a non-eagle. Thus, each rate is based on a different portion of the analyzed data (false-negative and false-positive rates will not sum to 1). We compared correct and incorrect error rates directly (without statistics) because these were comparisons of all data for each time period (without sampling).

**Table 1:** Time periods that identified major changes to the IdentiFlight system at the Manzana Wind Power Project, California, USA from 21 June 2018 to 20 June 2019. See S2 File for detailed description of system changes.

Time period	Start Date	End Date	Number of Tracks	Number of Records	Key Characteristics of System Configuration
Original Configuration	21 Jun 2018	31 Aug 2018	6,794	66,931	Feature-based Identification
IdentiFlight System Update	8 Sep 2018	19 Oct 2018	4,406	43,317	Neural Network Implementation
Equipment Issue	20 Oct 2018	26 Feb 2019	16,134	178,952	Lens Seal Failure + Neural Network
Equipment Replacement	27 Feb 2019	25 Apr 2019*	13,071	170,165	Lens Seal Replacement + Neural Network + Feature-based Override of Neural Network
Neural Network Upgrade	26 Apr 2019	20 Jun 2019	13,077	152,172	Neural Network Upgrade + Feature-based Override Removed

* Excluding dates 2–19 Apr 2019 when clock of the IdentiFlight System became uncalibrated.

### Assessment of curtailment orders

The IdentiFlight system recorded virtual curtailment orders for targets that it both identified as an eagle and determined was at risk of colliding with a wind turbine according to a curtailment prescription (Fig 1). These records included start and end times of individual curtailment orders, the duration of the curtailment, the track ID for which the curtailment was initiated, identification of the turbine that would have been curtailed, and the IdentiFlight camera tower that recorded the eagle track. We then determined the number,duration, and closest distance to wind turbines for curtailment orders both for targets correctly identified by the IdentiFlight system as eagles and for targets incorrectly identified as eagles (false-positive errors). To facilitate comparisons among time periods, which differed in duration, we standardized curtailment orders by number and duration (hrs) per week. We also summarized curtailment orders among turbines that were either fully covered (8) or partially covered (13) by the IdentiFlight system (Fig 2). We summarized closest distances of targets to wind turbines as remained far (>100 m) from, came near (0–100 m) but did not enter RSZs or entered RSZs.

## Results

### Identification accuracy

During this study, the IdentiFlight system archived a total of 611,537 records comprising 53,482 unique tracks, from both towers combined. The system identified 118,460 (19.4%) targets as eagles and 493,077 (80.6%) targets as non-eagles. Raptor biologists determined that 44,325 (7.2%) records were eagles, 566,081 (92.6%) records were non-eagles, and 1,131 (0.2%) records had no image to identify (Table 2). Overall, the IdentiFlight system correctly classified 77.0% (34,124 of 44,325) of targets that were determined to be eagles and 85.2% (482,295 of 566,081) of targets that were determined to be non-eagles.

**Table 2:** Counts of correct (bold) and incorrect identifications of eagles and non-eagles by the IdentiFlight system for each of five time periods, at the Manzana Wind Power Project, California, USA. Incorrect identifications included targets in which eagles were misidentified as non-eagles (false-negative errors, underlined) and non-eagles misidentified as eagles (false-positive errors, italics). Instances in which raptor biologists had no image to review were omitted from the analysis.

Time period	Raptor biologist determination	Number of records	IdentiFlight identification	
			Eagle	Non-eagle
Original configuration	Eagle	4,369	**3,975**	394
	Non-eagle	62,562	*9*,*362*	**53,200**
	No Image	0	0	0
IdentiFlight system update	Eagle	3,110	**2,134**	976
	Non-eagle	40,025	*1*,*715*	**38,310**
	No Image	182	82	100
Equipment issue	Eagle	16,725	**10,925**	5,800
	Non-eagle	161,710	*14*,*000*	**147,710**
	No Image	517	158	359
Equipment replacement	Eagle	16,861	**14,158**	2,703
	Non-eagle	152,894	*35*,*090*	**117,804**
	No Image	410	306	104
Neural network upgrade	Eagle	3,260	**2,932**	328
	Non-eagle	148,890	*23*,*619*	**125,271**
	No Image	22	4	18
All time periods (Total)	Eagle	44,325	**34,124**	10,201
	Non-eagle	566,081	*83*,*786*	**482,295**
	No Image	1,131	550	581

**Fig 3:**
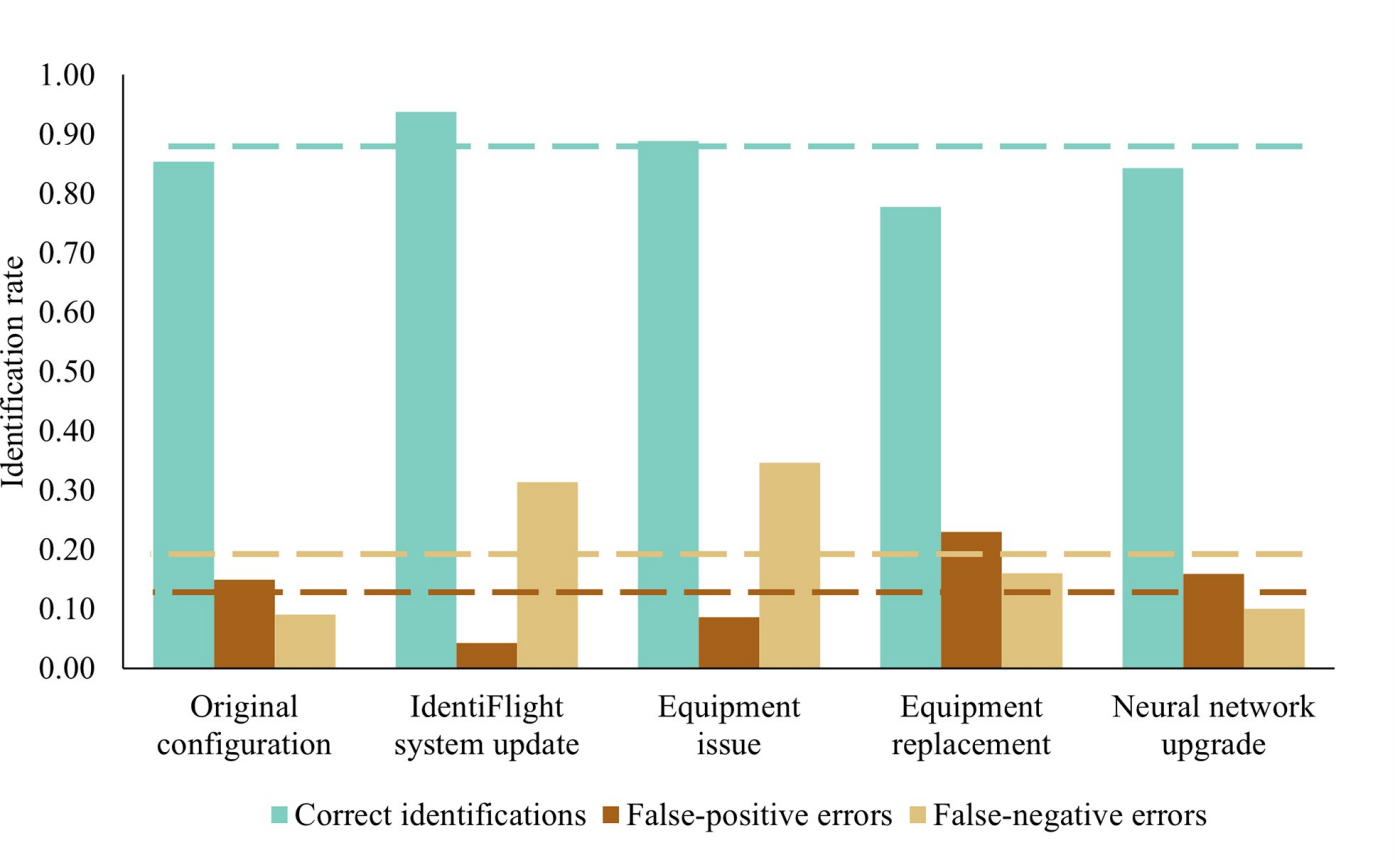
Correct and incorrect identification rates by the IdentiFlight system during each of the five time periods over which the system changed (see Table 1 and S2 File). Average rates are shown by dashed lines. Each rate is based on a different subset of the data; correct identifications apply to all records, false-negative errors apply only to eagles and false-positive errors apply only to non-eagles, as determined by raptor biologists.

Rates of correct and incorrect identifications differed among the time periods that defined changes to the IdentiFlight system at the Manzana Wind Power Project (Fig 3). Among time periods, IdentiFlight correctly identified 86% (sd = 5.9%) of records, misidentified 13% (sd = 7.1%) of eagles as non-eagles (false-negative error) and misidentified 20% (sd = 12.0%) of non-eagles as eagles (false-positive error). The rate of correct identifications was highest after the neural network was added to the identification algorithm (IdentiFlight system update), although for this time period rates of false-positive errors decreased to their lowest level while rates of false-negative errors increased (Fig 3). Identification rates changed only slightly during the time period when gasket failures caused the outer viewports of the stereo cameras to become occluded by an oily residue (equipment issue time period), although false-negative errors were at their highest level. During the equipment replacement time period, when viewports were replaced and a neural-network override was implemented, the correct identification rate was at its lowest, while false-positive errors increased to their highest level. During the last time period, when the neural network was upgraded, error rates returned to levels slightly higher than that of the original configuration of the IdentiFlight system at the Manzana Wind Power Project.

### Assessment of curtailment orders

The IdentiFlight system archived 6,677 curtailment orders, for 21 turbines (8 fully covered and 13 partially covered; see Fig 2). Of these curtailment orders, we paired 6,289 with target identities as determined by raptor biologists. We excluded the 388 curtailment orders from analysis because target images were not available.

Of the 6,289 curtailment orders that we analyzed, 850 orders were issued for eagle targets that were correctly identified by the IdentiFlight system for a total duration of virtual curtailments of 53.6 hrs. The mean duration of orders for eagle curtailments was 0.06 hrs (3.6 mins; sd = 0.02 hrs). The remaining 5,439 curtailment orders were issued for targets that were misidentified as eagles (false-positive errors) with a total duration of virtual curtailments of 301.3 hrs (duration mean = 0.06 hrs, sd = 0.01). The IdentiFlight system issued 13 curtailment orders for eagles that entered RSZs of turbines and 6276 curtailment orders for either eagles that did not enter RSZs or for non-eagles (Table 3). Overall, 98% of eagles and 69% of non-eagles remained >100 m from RSZs of turbines.

**Table 3:** Number and rates (no/wk) of virtual curtailments for targets that were correctly identified as eagles and incorrectly identified as non-eagles by the IdentiFlight system for each of five time periods, at the Manzana Wind Power Project, California, USA.

Time Period	Target Distance to RSZ of Turbine					
	>100 m		0-100m		Within	
	No	No/wk	No	No/wk	No	No/wk
	Eagles					
Original configuration	116	11.4	12	1.2	1	0.1
IdentiFlight system update	47	8.0	10	1.7	1	0.2
Equipment issue	318	17.3	40	2.2	10	0.5
Equipment replacement	217	40.0	10	1.8	1	0.2
Neural network upgrade	66	8.4	1	0.1	0	0.0
	Non-eagles					
Original configuration	235	23.2	74	7.3	22	2.2
IdentiFlight system update	87	14.9	66	11.3	18	3.1
Equipment issue	931	50.5	413	22.4	112	6.1
Equipment replacement	1045	192.5	240	44.2	59	10.9
Neural network upgrade	1462	186.1	588	74.8	87	11.1

**Fig 4:**
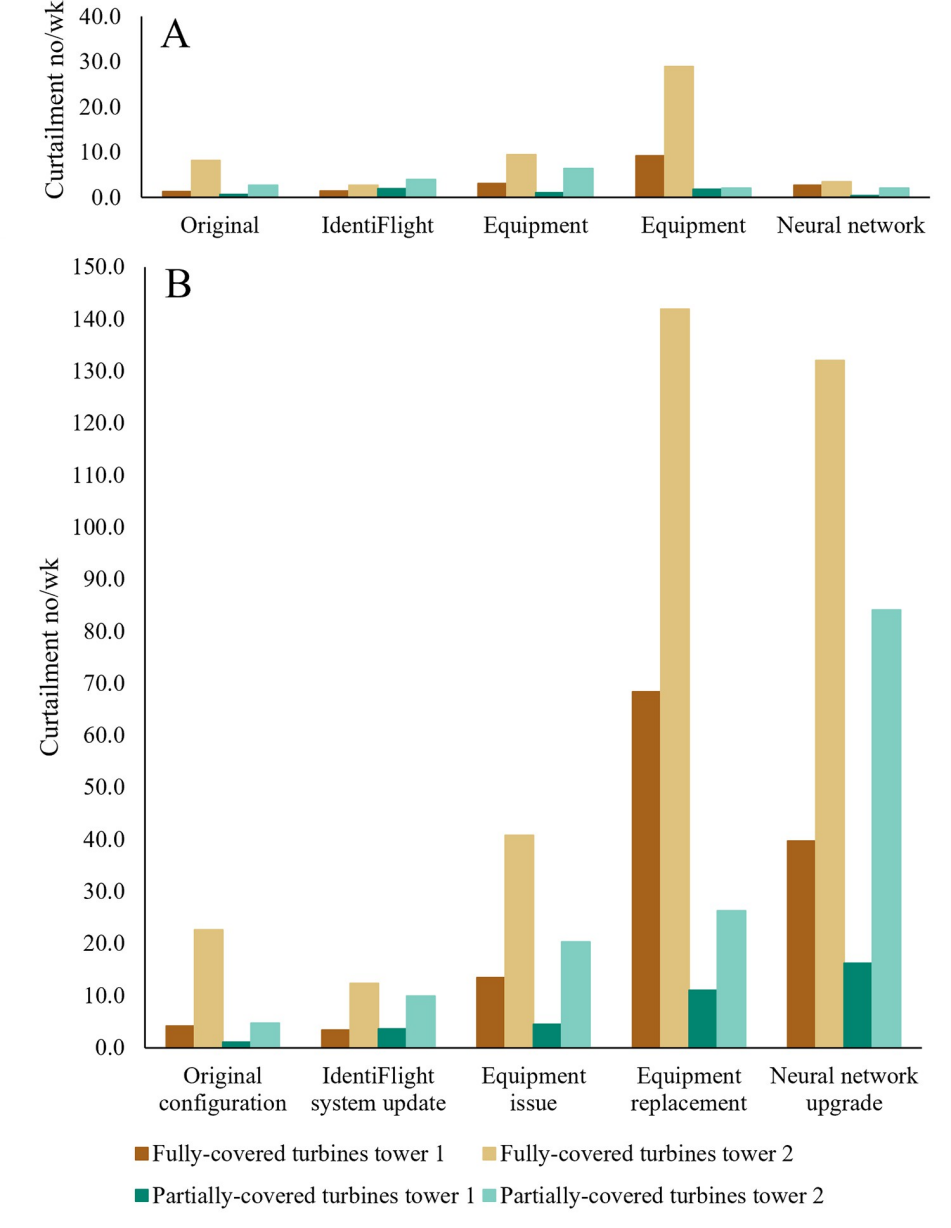
Rates of virtual curtailments (no/wk) issued by the IdentiFlight system for targets A) that it correctly identified as eagles and B) that it misidentified as eagles at the Manzana Wind Power Project, California, USA over time periods in which the system changed June 2018 to June 2019.

Virtual curtailment rates were higher for non-eagle targets (false-positive errors) than for eagle targets (correct identification) in all time periods (Fig 4). During the Equipment Replacement time period, curtailments for eagles peaked for turbines fully covered by the IdentiFlight system but the peak for partially covered turbines was during the Equipment Issue time period. Curtailments orders for non-eagles at fully covered turbines also peaked during the Equipment Replacement time period but for partially covered turbines, the peak was during the Neural Network Upgrade time period.

## Discussion

The IdentiFlight system installed at Manzana Wind Power Project correctly identified 77% of eagles and 85% of non-eagles; however, such accuracy did not directly translate into similar curtailment accuracy for eagle and non-eagle targets. Curtailment orders for non-eagle targets occurred 6 times as frequently as for eagle targets (Fig 4). The IdentiFlight system, during the year-long assessment, recorded 850 (53.6 hrs) virtual curtailments for intended targets and 5,439 (301.3 hrs) virtual curtailments for unintended targets across 8 turbines considered fully covered and 13 turbines considered partially covered by the system. Had the IdentiFlight system issued actual curtailments, their numbers and duration could have differed from virtual curtailments due to either conditioning of or responses to changes in the speed of wind turbines for both eagles and non-eagles. Ineffective curtailments were primarily due to the greater abundance of non-eagle targets, which were primarily common ravens (*Corvus corax*) misidentified as eagles at the study site. A winter study found that common ravens are more than 140 times more abundant than are golden eagles in the deserts of southern California, USA [12]. Ultimately, rates of ineffective curtailments (unintended targets) were dependent upon identification accuracy and the relative abundance of different species at a project site.

For a given curtailment to be effective, the target must be both at risk of colliding with a wind turbine and an intended target of the system. If entry into the RSZ is used to define risk of colliding with a turbine, <1% of curtailments ordered by the IdentiFlight system fit this definition. Expanding risk to include eagles that come within 100 m of the RSZ (including red cylinder Fig 1), 10% of curtailments were effective. The low rates for effective curtailments identifies two areas where the IdentiFlight system could be improved; better prediction of an eagle entering the RSZ and improved discrimination between intended and unintended targets.

At the Manzana Wind Power Project, changes to the IdentiFlight system had substantial consequences for error rates in identification of targets (Fig 3). Specifically, the first implementation of the neural network, during the IdentiFlight system update time period, resulted in a higher rate of false-negative errors (increased by 0.21) but lower rate of false-positive errors (decreased by 0.11; Fig 3). This initial implementation of the neural network was focused on reducing errors from misidentifying common ravens as eagles (false-positive errors), which occurred at the expense of misidentifying eagles as non-eagles (false-negative errors). Implementation of the neural network override (equipment replacement time period), intended to reduce false-negative errors, functioned as expected. Somewhat surprisingly, the error rates during the equipment issue time period increased marginally (by 0.03–0.04) compared to the prior time period, which means that the equipment failure had a minimal impact on error rates. Finally, during the last time period, neural network upgrade–when the number of images used in the neural network was increased, error rates returned to levels that were slightly higher (by 0.01) than for the original configuration. Changes to the AI algorithms of the IdentiFlight system throughout this study resulted in substantial changes to misidentification rates. The feature-based identification algorithm initially used by the system performed as well or better than the more complex combination of feature-based and neural network algorithms used at the end of the study. These patterns in error rates also illustrate a trade-off between false-negative errors and false-positive errors, which in turn suggests that costs for a wind-power operator may be adjusted according to their own risk tolerance levels.

Curtailment effectiveness of an AI-based system was influenced by identification accuracy and relative abundance of a species of bird that was an unintended-target but is also likely to be influenced by additional factors. The time required to curtail a wind turbine could influence effectiveness by increasing the distance at which curtailments must be issued to be effective. At another North American wind project, where turbines could be curtailed as quickly as 20 s, this system was shown to reduce eagle collisions by 82–85% [7, 8]. However, the average time required to curtail turbines at Manzana Wind Power Project is approximately 60 s (A.E.P. unpublished data). The effectiveness of the system is also likely influenced by the setting in which the system is placed. At the other North American site, data recorded by an IdentiFlight system showed turbine-specific patterns of entry into RSZ [13], illustrating that collision risk varies within a wind-power project. It is also likely that collision risk varies among wind-power projects; however, additional research is needed to determine if the performance of AI-based systems differs among wind-power projects and differs as IdentiFlight refines their AI-based algorithms, including target identification and target trajectory toward turbines.

The balance between benefits and costs, in terms of reducing risk of collisions between target species and turbines and in terms of price of the IDF System, reduced energy generation, and turbine wear and maintenance, may depend upon the specific characteristics of each wind energy project. These characteristics include the biological setting (e.g., avian community and relative risk of target species colliding with turbines), speed at which operators can curtail turbines, and the objectives of the operator when considering the IDF System.

## Supporting information

S1 File Description of the IdentiFlight system. (DOCX)

S2 File Changes to the IdentiFlight system at Manzana Wind Power Project, California, USA. (DOCX)
