# Supplementary material for: Effectiveness of an artificial intelligence-based system to curtail wind turbines to reduce eagle collisions
Source: PLoS One. 2023 Jan 26;18(1):e0278754. doi: 10.1371/journal.pone.0278754 (PMC9879396; doi:10.1371/journal.pone.0278754)
Supplement: S1 File — (DOCX) [file pone.0278754.s001.docx]

Supporting Information 1. Description of the IdentiFlight System

The IdentiFlight (IDF) System is designed to detect, identify and track large avian species (targets) up to 1000 m from IDF Towers. An IDF System at a wind power project consists of one or more IdentiFlight Towers (also called Inspectors) interconnected with an IdentiFlight Base Station. IDF Towers provide visual coverage for selected wind turbines. Each IDF Tower relays key data to a centrally located Base Station (computer system), where decisions are made according to a predetermined curtailment prescription regarding which turbines pose a risk to a given protected species of bird and should be directed to shut down until the bird clears the area of collision risk.

Each IDF Tower includes two types of cameras mounted on a 7-10 m high tower. A ring of eight wide-field-of-view (WFOV) cameras are mounted below one set of high-resolution stereo (HRS) cameras affixed to a pan and tilt unit (PTU) (Figure S1.1). The eight WFOV cameras are fixed in position with each one scanning a “detection zone” that is 65.7° vertically and 55° horizontally (Figure S1.2). The horizontal volume of space scanned by individual WFOV cameras overlap such that the set of eight cameras scan the volume of space 360° around an IDF Tower (Figure S1.2), although there is a “detection occluded zone” directly above and a “below detection zone” below each tower. The vertical orientation of WFOV cameras can be adjusted individually to match the terrain where each tower is installed. The WFOV cameras at Manzana Wind Power Project were installed such that they pointed at slightly different angles from horizontal (Table S1.1). The WFOV cameras detect moving objects (targets) represented by enough pixels within an image such that the object might be a large avian species of interest while ignoring other moving objects (image patterns) that are not of interest. After an image of interest is detected by the WFOV cameras, the IDF System directs HRS cameras to begin tracking it.


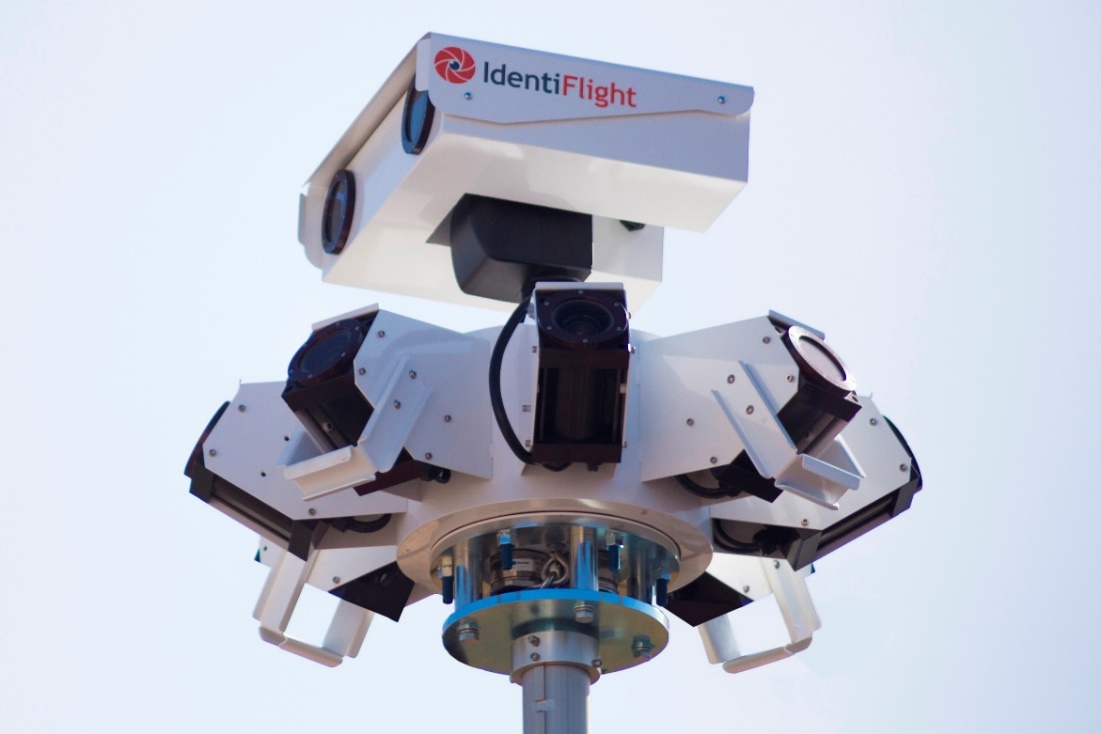


Figure S1.1. The IdentiFlight system includes high resolution stereo cameras mounted on a pan and tilt unit above eight wide-field-of-view cameras (Image Credit: IdentiFlight).


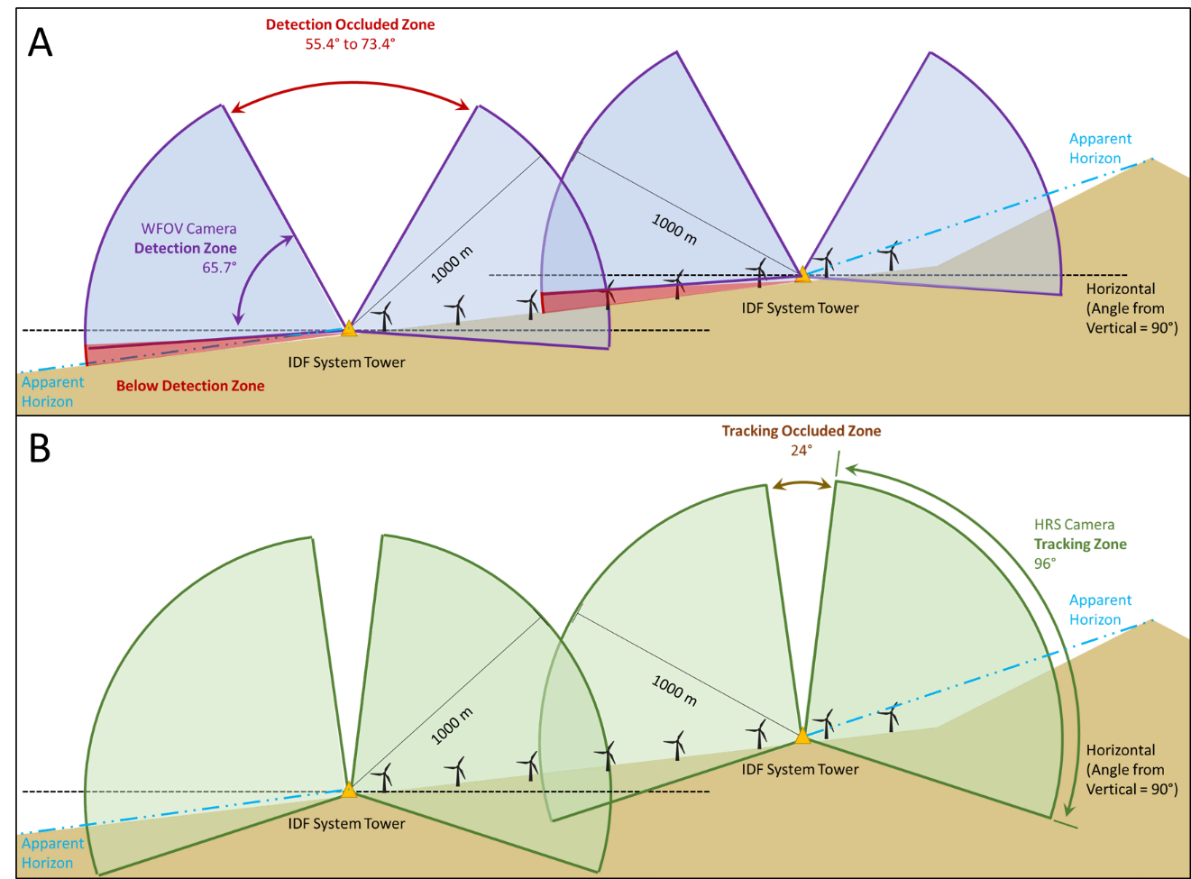


Figure S1.2. illustration of vertical space around turbines scanned by IdentiFlight system cameras (yellow triangles; modified from image created by IdentiFlight). A) Wide-field-of-view (WFOV) cameras scan the detection zone (blue shading) over 65.7° vertically, ranging from 3.35° - 11.35° below horizontal to 54.3° - 62.35° above horizontal at Manzana Wind Power Project. The apparent horizon is illustrated from each tower outward (light blue dash-dot lines). The volume of space not scanned by WFOV cameras include the detection occluded zone (red arrow) above towers and below detection zone (red shading) below towers. B) High resolution stereo (HRS) cameras track over 96° vertically (the tracking zone in green), from 18° below horizontal to 78° above horizontal. The volume not tracked above the horizon is the tracking occluded zone (brown). Ground is depicted in tan.

The HRS cameras can track a target 360 degrees horizontally and anywhere between 18 degrees below horizontal (level) and 83 degrees above horizontal, with areas above this volume falling within the “tracking occlusion zone”. HRS cameras take high-resolution stereo images of targets at a rate of ten frames per s, with the system recording approximately one per s. Stereo images from the HRS cameras are used to estimate the distance from the tower to the target.

Targets are identified by the IdentiFlight System through two artificial intelligence processes (algorithms). The first algorithm uses the feature-based attributes of targets (e.g., wingspan based on a partial side view as determined by comparing target length along the major axis with length along the minor axis of an image). Feature-based attributes of targets (e.g., body length, wind span, wing posture, color composition) are estimated based on distances to targets and WFOV camera images. Estimated feature sizes of a target are compared to feature sets within a classification table to determine the target identification and an associated confidence statistic. The confidence statistic is a measure of how well the identification matches the IDF System’s library of image attributes. The second algorithm uses a convolutional neural network to identify targets. IdentiFlight trains neural networks with thousands of images of large birds that they obtain at many project sites, that have been identified to species, and that were taken under atmospheric conditions that are likely to occur at a given project site. Neural network identifications also include confidence statistics. In some situations, the neural network identification can be overridden by feature-based attributes (e.g., wingspan estimate) to bias identification, thus protection through curtailment orders, toward specific protected species (e.g., golden eagles). For example, targets with wingspans larger than 1.8 m could be automatically identified as eagles. Such an override could be included as part of the curtailment prescription (described below) or to adjust for identification errors.

Table S1.1. Vertical and horizontal orientation of each of the eight wide-field-of-view cameras installed at the Manzana Wind Power Project 21 June 2018 to 20 June 2019.

| IDF Tower No. | WFOV Camera No. | Heading Angle Center (°) | Heading Coverage | | Tilt Angle Center (°) | Tilt Coverage | |
| --- | --- | --- | --- | --- | --- | --- | --- |
|  |  |  | Left Heading Angle (°) | Right Heading Angle (°) |  | Below Horizontal Tilt Angle (°) | Above Horizontal Tilt Angle (°) |
| 1 | 0 | 10.5 | 0 | 33 | 29.5 | -3.35 | 62.35 |
| 1 | 1 | 55.5 | 33 | 78 | 29.5 | -3.35 | 62.35 |
| 1 | 2 | 100.5 | 78 | 123 | 29.5 | -3.35 | 62.35 |
| 1 | 3 | 145.5 | 123 | 168 | 21.5 | -11.35 | 54.35 |
| 1 | 4 | 190.5 | 168 | 213 | 21.5 | -11.35 | 54.35 |
| 1 | 5 | 235.5 | 213 | 258 | 21.5 | -11.35 | 54.35 |
| 1 | 6 | 280.5 | 258 | 303 | 21.5 | -11.35 | 54.35 |
| 1 | 7 | 325.5 | 303 | 348 | 29.5 | -3.35 | 62.35 |
| 2 | 0 | 347 | 0 | 9.5 | 27.5 | -5.35 | 60.35 |
| 2 | 1 | 32 | 9.5 | 54.5 | 27.5 | -5.35 | 60.35 |
| 2 | 2 | 77 | 54.5 | 99.5 | 27.5 | -5.35 | 60.35 |
| 2 | 3 | 122 | 99.5 | 144.5 | 21.5 | -11.35 | 54.35 |
| 2 | 4 | 167 | 144.5 | 189.5 | 21.5 | -11.35 | 54.35 |
| 2 | 5 | 212 | 189.5 | 234.5 | 21.5 | -11.35 | 54.35 |
| 2 | 6 | 257 | 234.5 | 279.5 | 21.5 | -11.35 | 54.35 |
| 2 | 7 | 302 | 279.5 | 324.5 | 27.5 | -5.35 | 60.35 |

The IDF System attempts to detect and identify protected birds at sufficient distances from a wind turbine to initiate shutdown (curtailment) of that turbine to reduce the risk of a collision. The curtailment prescription resides on the IDF Base Station, where signals can be sent automatically to the Supervisory Control and Data Acquisition (SCADA) system of the wind power project to control individual turbines. The distance from a turbine at which curtailment is initiated depends on many things including the terrain, avian species present, the bird’s flight vector, the rotor slowdown characteristics of the turbine, where the bird was first detected, the confidence with which the IDF tower identified the species, etc.

Curtailment prescriptions are site-specific and IdentiFlight intends them to both evolve and be set in conjunction with the operators of a wind power project. An illustration of the curtailment prescription used at the Manzana Wind Power Project is shown in Figure 1 (of main text). This prescription orders curtailments when one of two sets of conditions are met. The first set of conditions include that a target has been identified as an eagle with > 89% confidence, that the target is located below 400 m above ground level (AGL) and within 350 m of a wind turbine, and that the estimated time to collision for a target is ≤ 60 sec. The second set of conditions include that the confidence that the target is an eagle >89% and that the target is ≤ 200 m AGL and within 200 m of a wind turbine. Curtailments are released 3 min after the above conditions are no longer met.

IdentiFlight makes several recommendations relative to installation and operation of the IDF System (personal communications, T. Hiester, IdentiFlight). IDF Towers should be installed such that occlusion zones for each tower should fall within 300-700 m (distance of best detection) of another IDF Tower to prevent gaps in coverage for the IDF System. Ideal spacing of IDF Towers places towers such that turbines of interest are fully covered. Individual turbines are considered fully covered by the IDF System when the 1000-m detection distance for IDF towers encompasses a 600-m buffer around individual turbines. This 600-m buffer is based on preliminary estimates of eagle flight and times required to curtail a turbine to rotational speeds that are slow enough not to represent a risk of collisions for eagles. IdentiFlight assumed a flight speed of 20 m/s for eagles, thus an eagle flying directly toward a turbine will reach the turbine center from the 600 m distance in 30 sec. This 30-sec time would allow for a turbine to be curtailed to 2 rotations per minute, based on characteristics of Siemens wind turbines installed at Top of The World Wind power project in Wyoming (see McClure et al. 2018).

Some of these recommendations illustrate several assumptions that IdentiFlight has made. First, golden eagles do not directly approach wind turbines at speeds greater than 20 m/s. Second, 2 rotations per minute is safe and would minimize the risk of eagle-turbine collisions. Third, 30 seconds is enough time to safely curtail wind turbines. Wind power project operators should consider each of these relative to project-specific conditions that may not match these assumptions. Changes to either the configuration of the IDF System or the details of the curtailment prescription can be modified to accommodate modifications of these assumptions to better match the specific characteristics of the site, to better match the risk tolerance of a given wind project operator, and to better match the characteristics of protected species at risk of colliding with wind turbines.
