# Supplementary material for: Effectiveness of an artificial intelligence-based system to curtail wind turbines to reduce eagle collisions
Source: PLoS One. 2023 Jan 26;18(1):e0278754. doi: 10.1371/journal.pone.0278754 (PMC9879396; doi:10.1371/journal.pone.0278754)
Supplement: S2 File — (DOCX) [file pone.0278754.s002.docx]

Supporting Information 2. Changes to the IdentiFlight System at Manzana Wind Power Project, California, USA.

From 21 June 2018 through the 20 June 2019, the IdentiFlight system went through a series of major changes that included modifications of the identification algorithms (feature-based and neural network), equipment issues and equipment changes (see Table 1 in the main text). Each of these changes were intended to reduce identification errors; therefore, we used the dates bounding these changes to define 5 time periods. We analyzed data separately for each time period. The IdentiFlight system also went through additional changes and maintenance that did not directly affect identification of targets. (see Table S2.1).

The first time period was based on the Original Configuration of the IdentiFlight system. The system identified targets during this bin using only the feature-based identification algorithm, which identified eagles through measurements of a given target’s wingspan and body size (see Supporting Information 1).

The Original Configuration was updated on 1 September 2018 by the addition of a neural network. The neural network was added to compensate for misidentification of common ravens (*Corvus corax*), a seasonally abundant species at the Manzana Wind Power Project, as eagles. The neural network is an artificial intelligence system that was trained on images of large birds, which were identified to species by IdentiFlight experts, and were taken by IdentiFlight systems installed at the Manzana Wind Power Project and elsewhere under a variety of atmospheric conditions. On 8 September 2018 the operating system for the whole IdentiFlight system was updated. These changes resulted in a brief 7-day time window in which the configuration was unique. We excluded data (9,641 records organized into 831 tracks) collected during these dates (1-7 September 2019) from analysis because the system did not collect adequate data to make a separate evaluation of that configuration.

The second time period, IdentiFlight system update, included the time period from 8 September 2018 to 19 October 2018. This time period represented two changes to the IdentiFlight system; the addition of the neural network identification algorithm and an update to the computer operating system.

The third time period was defined as equipment issue. During the time period of 20 October 2018 to 26 February 2019, certain gaskets on the HRS cameras failed. These gaskets sealed the viewports, which are the outermost round covers for the HRS cameras (see Figure S1.1). The gasket failures created an oily residue on the viewports, which caused a degradation of the quality of images recorded by the HRS cameras.

The fourth time period was designated as equipment replacement, which occurred on the 27 February 2019. During this time period, a neural network override was also implemented. This override was a secondary check to the neural network identification algorithm in which the IdentiFlight system classified the target as an eagle if measurements of the wingspan exceeded a predetermined minimum threshold (1.8m). This effectively reduced the size in which targets were automatically classified as eagles. The Equipment Replacement time period ended on 25 April 2019, when the last system change was made. From 2 April 2019 to 19 April 2019 the connection of the IdentiFlight system to the internet was lost, which resulted in a loss of calibration between the IdentiFlight system clock and an atomic clock. We excluded this uncalibrated data from analyses.

The fifth time period began on 26 April 2019 after a neural network upgrade was implemented. This upgrade to the neural network was based on an expanded set of images of large birds that were identified by IdentiFlight experts (see Supporting Information 1) and used by the neural network for target identification. This time period included data collected through the end of the study.

In addition to the major changes described above, the IdentiFlight system also underwent several minor changes and maintenance procedures (Table S2.1).

Table S2.1. Changes and maintenance procedures made to the IdentiFlight system at Manzana Wind Power Project, California, USA during the study from 21 June 2018 through the 20 June 2019.

| IDF System Component | Date Start^1^ | Date End | Details |
| --- | --- | --- | --- |
| Tower 2 |  | 6/29/2018 | Stereo Distance Calibration |
| Tower 1 |  | 6/29/2018 | Stereo Distance Calibration |
| Tower 2 | 7/3/2018 | 7/6/2018 | Stereo Board power failure |
| Tower 2 |  | 7/8/2018 | Stereo Distance Calibration |
| Tower 1 |  | 7/8/2018 | Stereo Distance Calibration |
| Tower 2 |  | 7/12/2018 | Stereo Distance Calibration |
| Tower 2 |  | 7/18/2018 | Software Upgrade |
| Tower 1 |  | 7/18/2018 | Software Update to Implement Keep Out Zone so PTU Doesn't Park Staring at Customer's House |
| Tower 1 |  | 8/2/2018 | Stereo Distance Calibration |
| Towers 1, 2 |  | 8/27/2018 | Updated PTU Homing Commands |
| Towers 1, 2 |  | 8/31/2018 | Initial Neural Network Went Live, Continuous Pan Update |
| Tower 1 |  | 8/31/2018 | Neural Network Software Update |
| Towers 1, 2 |  | 9/7/2018 | OS Image Update to 1.0.8 |
| Base Station |  | 9/10/2018 | Condor Added to Base Station as Protected Species |
| Towers 1, 2 |  | 9/19/2018 | Software Upgrade to Detect SQL Connection Failures and Retry. This Also Addressed a Potential Timestamping Issue with the Base Station. |
| Tower 1 |  | 9/19/2018 | Software Update (SQL patch) |
| Tower 1 |  | 10/11/2018 | Stereo Distance Calibration |
| Tower 1 |  | 10/16/2018 | Stereo Distance Calibration |
| Towers 1, 2 |  | 11/13/2018 | Neural Network with Condor Went Live. PTU Control Algorithm Improvements |
| Towers 1, 2 |  | 11/16/2018 | Neural Network Reverted to Original Implementation from 8/31/2018 |
| Tower 1 | 2/26/2019 | 2/26/2019 | Lens Cleaning/ Installed Spare Hard-Drive to Convert DB Storage Engine |
| Tower 2 | 2/26/2019 | 2/26/2019 | Lens Cleaning/ Installed Spare Hard-Drive to Convert DB Storage Engine |
| Base Station | 2/26/2019 | 2/26/2019 | Installed Hyper-V to Run NMS VM |
| Met-1 | 4/1/2019 |  | Met-1 Tower was Noticed to be Measuring Long. Went to Calibrate However Target was Gone. Sent New Target to Site |
| Base Station | 4/3/2019 | 4/19/2019 | Base Station Maintenance (Replaced Hardware) |
| Towers 1, 2 |  | 4/25/2019 | Neural Network 2.4_6M Upgrade |
| Base Station/Met-1 | 4/28/2019 | 4/29/2019 | NTP Sync Issue (NTP Time Drift). Needed to Open UDP Port on Base Station. (It is Possible Curtailments May Not Have Been Triggered During This Time Frame |
| Tower 2 |  | 5/13/2019 | Stereo Distance Calibration |
| Tower 1 | 5/13/2019 | 5/16/2019 | Stereo Distance Calibration (Single Point Calibration) |
| Tower 1 |  | 5/21/2019 | Full Stereo Distance Calibration |
| Tower 1 |  | 7/3/2019 | Full Stereo Distance Calibration |
| Tower 2 |  | 8/8/2019 | Full Stereo Distance Calibration |
| Tower 1 |  | 8/8/2019 | Stereo Distance Calibration |

^1^Changes without a start date were completed entirely on the end date.
